# Ramadan Fasting and NCDs-Example of the Diabetes

**DOI:** 10.3389/fnut.2022.787571

**Published:** 2022-03-02

**Authors:** Meriem Bencharif, Ibrahim Sersar, Maroua Bentaleb, Fatima Zohra Boutata, Youcef Benabbas

**Affiliations:** ^1^Institute of Nutrition, Food and Agro-Food Technologies (INATAA), University of Brother's Mentouri Constantine1 (UFMC1), Constantine, Algeria; ^2^Laboratory of Nutrition and Food Technology (LNTA), University of Brother's Mentouri Constantine1 (UFMC1), Constantine, Algeria; ^3^Laboratory of Food, Nutrition and Health (ALNUTS), University Salah Boubnider Constantine 3, Constantine, Algeria; ^4^Institute of Veterinary Sciences and Agronomic Sciences, Elhadj Lakhdar University, Batna, Algeria; ^5^Department of Internal Medicine, Hospital University, Constantine, Algeria

**Keywords:** Ramadan fasting, NCDs, diabetes, lifestyle habits, nutritional education, Muslim patient

## Abstract

Although Ramadan lasts only for 1 month each year, it can be accompanied by significant changes in: both energy and nutritional intake; in the diet composition; in the working hours; and the usual way of life. The majority of practitioners consume two meals, one after sunset (*Iftar*) and one before dawn (*Sohor*). During this month, it is also an opportunity to share a meal with family and friends, a period of highly intensified socialization. In parallel with the nutritional changes brought about by this unique pattern of fasting in Ramadan, other metabolic and physiological changes may occur, such as fluctuations in body weight and/or disturbance in the quantity and quality of the sleep-wake circadian rhythm. In the verses of the Qur'an, the exemption from fasting in certain situations such as illness is clearly stated. Despite this religious tolerance, many faithful who are eligible for the exemption observe the fast of Ramadan either for the spiritual aspect it provides by performing it, by religious guilt or to mark a normalization in the Muslim community for fear of the gaze of others. The world is experiencing an increase in the emergence of non-communicable diseases (NCDs); leading cause of the global mortality. Environmental and behavioral risk factors related to lifestyle, such as smoking, excessive alcohol consumption, unhealthy diet, and sedentarity have a causal association with NCDs. Other factors, such as genetic and physiological factors may also be associated (overweight, high blood pressure, dyslipidemia). Diabetes is one of the highest prevalent NCDs in the world and it continues increasing year by year. This chronic disease can lead to significant potential complications (degenerative, dermatological, and acute) to the patient's health. This requires an individual and appropriate care, both dietetic and therapeutic and over the long term will at best make it possible to sensitize the diabetic patient to the adverse effects related to his disease and thus improve its quality of life. Performing the Fast of Ramadan for a diabetic is a common situation. Diabetes is the only chronic disease widely studied in relation to Ramadan fasting. In the literature, many studies have investigated the effects of Ramadan intermittent fasting on diabetic patients. This article aims to provide a general overview and highlight if there are many effect of Ramadan fasting on diabetes, as an example of a NCDs.

## Introduction

Ramadan is one of the five pillars of Islam. It constitutes the month of fasting, a holy month for Muslims who have the duty to fast from sunrise to sunset. Ramadan lasts between 29 and 30 days depending on the lunar cycle, shifts from year to year and gradually changes from one season to another. This religious rite concerns all healthy Muslims in good health, and only those who are at risk of harm from fasting are exempted from this obligation such as people with non-communicable diseases (NCDs) or other disabling diseases. In the Muslim community, there is an intense desire to participate in fasting, even among those who are eligible for the religious exemption (1). Despite this, many diabetics fast despite the risk of complications and decompensation (2). Indeed, in the literature, several studies have been interested in fasting Ramadan related to diabetes as NCDs, and various works have been published on this subject around the world: in Lebanon (3), in Morocco (4), in France (5), in Tunisia (6), in Algeria (7, 8). The interest of this theme also lies in the fact that there are many millions of Muslims in the world and the proportion of people with diabetes who are diagnosed or not. Therefore, we have chosen in this literature review to provide a thematic overview of the problem ≪ Ramadan and diabetes ≫ by exposing research and recent advances in relation to behavioral and lifestyle changes (diet, sleep, physical activity), body composition (anthropometry), metabolic changes, the link with Covid-19 pandemic and the role of nutritional education, as well as the recommendations issued on this subject.

## Research Methods

**Figure 1:**
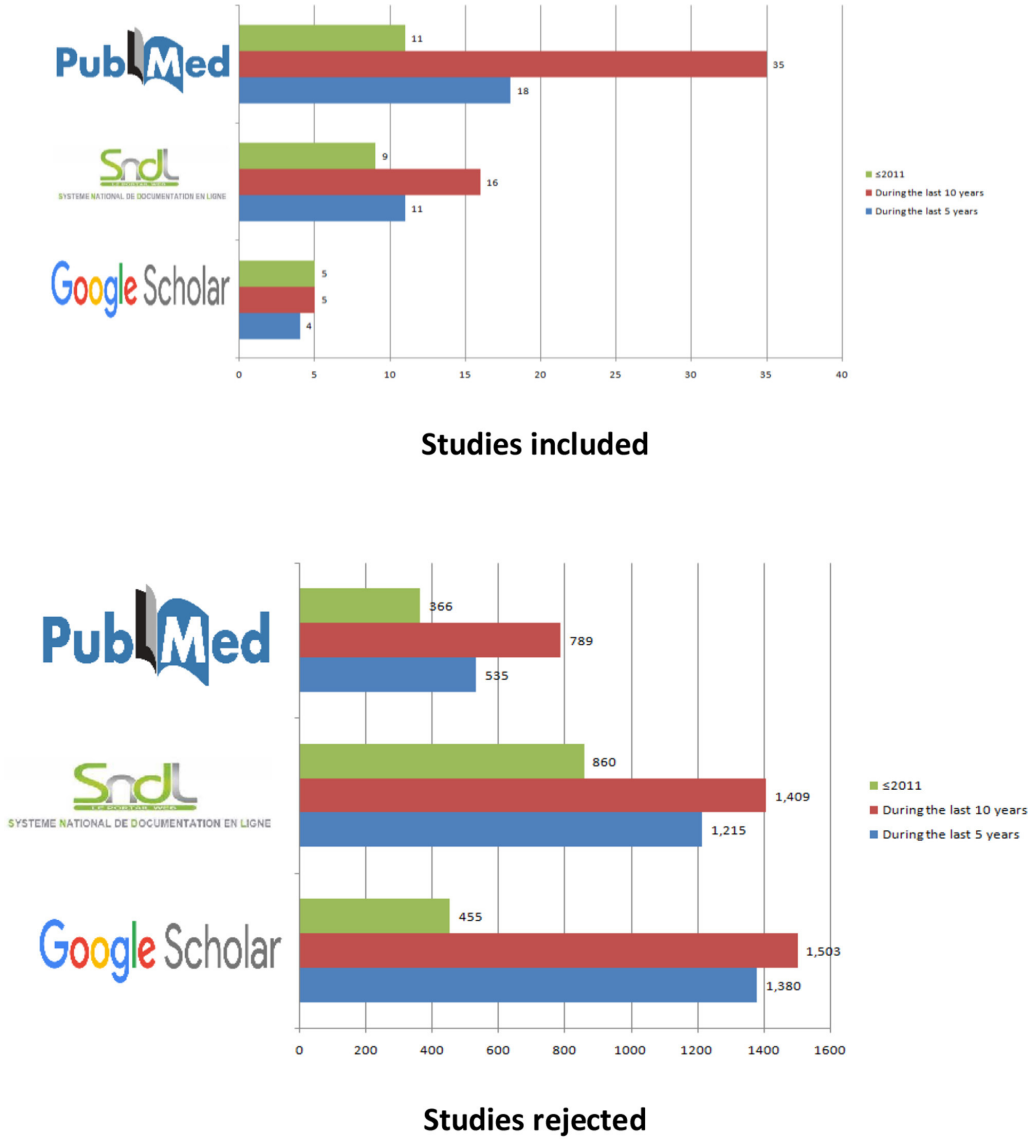
Studies included and rejected according to the literature search.

The PubMed and Google Scholar databases were searched. We also used a national online documentation system of the algerian university (SNDL.cerist). The key words used were “Ramadan fasting,” “fasting” and we have associated with each of these word “diabetes,” “NCDs,” “circadian rhythm,” “complications,” “dietary habits,” “physical activity,” “body composition,” “nutritionnal education.” We mainly took the recent and most relevant studies focused on human studies. The Figure 1 summarized the studies and works included and rejected according to the literature search carried out from these three databases.

## Eating Habits During Ramadan

Ramadan comes without transition, and practitioners move from one way of life to another overnight. The whole rhythm of everyday life is upset, thus reflecting a reversal of activities characterizing day and night. Normally, people eat during the day, and most socializing activities take place during the day. During Ramadan, the schedule of meals and activities is changed. These changes vary according to the seasons, geographical and socio-economic situations as well specific traditions of each country. In these conditions, the fasting body tries to adapt twice in the space of a month: at the beginning and end of Ramadan (9). The management of the diabetic patient during this month is of particular importance because of the crucial changes in lifestyle and diet both qualitatively and quantitatively. The change in eating habits has important implications for the physiological process, such as the impact on blood glucose control. People with diabetes must maintain a healthy and balanced diet during Ramadan. However, dietary intakes are exclusively nocturnal and are most often characterized by an overconsumption of carbohydrate products and fat preparations. These dietary changes have a direct impact on the health of both fasting (DMF) and non-fasting diabetics (DMNF) (10–12). A multicenter study was carried out in 13 cities in Algeria with 2,819 type 1 (T1DM) and type 2 (T2DM) diabetics showed that DMNF approached the topic of diet with their physician more than DMF (50.6 vs. 25.9%; P = 0.0000). Probably due to their diet during this month because, in fact, 94.6% of DMNF declared an increased consumption of carbohydrates foods, fat meals (91.6%), higher rations (90.5%) in the evening and heavy meals than usual (81.1%) (13). Throughout the month of Ramadan, there is an abundance and a variety of preparations on tables and can be sold in the market level, all rich in saturated fatty acids and carbohydrates (honey cakes, pastries, traditional dishes, etc.) that are difficult to resist; a self-control for both DMF and DMNF. Moreover, in another Algerian study carried out in 276 obese women with T2DM, showed that the fast-breaking meal (Iftar) alone provided 74% of the energy intake during the month of Ramadan, which is probably not without disadvantages (glycemic variations) in diabetic patients (7). A priori in the literature research results have shown either an increase in total daily energy intake associated with an increase in the intake of carbohydrates [Tunisian prospective study, (14)], or a decrease in these intakes with an increase in the intake of carbohydrates and saturated fatty acids [Moroccan study, (15)], or no significant change in energy intake [Ajman-UEA, (16)]. The heterogeneity of the results of the various studies can be attributed to the methods of estimating dietary intake, the survey season, socio-economic status, and other lifestyle variations of the different populations studied.

## Change in the Nycthemeral Cycle

Food deprivation, in general, is deemed to have an influence on a number of physiological functions (17). According to Khalfallah et al. (18), chronobiological inversion of nutritional habit is responsible of several functional disorders. Ramadan is a chronobiological desynchronization model which is characterized by a reversal of the foodboorne and waterboorne rhythm. The fasting of Ramadan has the particularity of constantly changing the synchronizers of the central clocks for a month. On healthy volunteers, a comparative study was carried out under identical observation conditions (Winter Ramadan and summer Ramadan). Researchers found that sleep debt could accumulate without compensation through daytime naps and that Ramadan alters the circadian organization of the sleep-wake rhythm, and this, most importantly, in summer when the photoperiod is the longest (19). Sleep can appear simply as a state of rest. It, nevertheless, constitutes a complex physiological state, necessary for the survival of the organism, characterized by different phases of deep sleep (20). The rhythm of life imposed by the month of Ramadan systematically leads to a decrease in the duration in sleep time. In a study of 2,708 diabetics (21), a sleep-wake cycle disturbance was observed, where sleep decreased during the month of Ramadan, while napping increased (*p* < 0.05). The results of another study carried out on 1,266 diabetic patients showed an average sleep duration between 5 to 8 h in 70.3% of T2DM and 65.5% of T1DM. The same results both for the DMF and DMNF (22). In an epidemiological study related to the sleep-wake rhythm during Ramadan, researchers found a significant increase in sleep latency, intra sleep warkining and daytime naps. These disturbances appeared from the first week of fasting, persisted throughout the month, and disappeared after the post-Ramadan period. This sleep-wake rhythm disturbances also persisted on weekends (23).

## Physical Activity Practice

Physical activity can help people with diabetes achieve a variety of goals, like improving their cardiorespiratory health; increasing their physical endurance; better controlling their blood glucose levels; reducing their insulin resistance; improving their lipid profile; lowering their blood pressure; and maintaining weight loss (24). Sedentary lifestyle in an individual can increase the risk of early mortality (25). In patients with T2DM, regular physical activity reduces the risk of all-cause mortality (between −30 and −40%), as well as cardiovascular mortality (−25 to −40%) (26). Indeed, the study of Kooiman et al. (27) in people with T2DM, showed an increase in the physical activity that was correlated with decreased levels of glycated haemoglobin (HbA1c) and blood glucose and decreased cardiovascular complications. The continuation of religious practices, as well as the disturbances of the circadian rhythm that are associated with the fasting of Ramadan, such as the perceived feeling of subjective fatigue, drowsiness, thirst and/or even mood swings, can lead to a significant decrease in the level of physical activity of Muslims (28). Results from a Moroccan study on healthy athletic adults showed a significant reduction in physical performance and a significant effect of Ramadan fasting on blood glucose and memory (29). Paradoxically, the results of a study on Algerian basketball players from a sports club showed no variations in training during the month of Ramadan and no significant effect on sports activity (30). According to Ghrici (31), the practice of moderate sport promotes physiological adaptation to fasting, since some changes during Ramadan are observed in sedentary people and not in athletes. This is the case with variations in blood glucose and triglycerides. Studies of substitution of sedentary time by physical activity (iso-temporal substitution) confirm the importance of replacing this sedentary time with physical activity, regardless of its intensity (25). During the month of Ramadan, physical activity levels must be maintained, whether for the general population or the sick population such as diabetics, but this is not always the case. In a study of Saudi diabetics, a high prevalence of physical inactivity and an increase in sedentary time were shown during Ramadan (32). While the work of Sfar et al. (33) showed a non-significant increase in physical activity in Tunisians with T2DM during Ramadan compared to the pre-Ramadan period. With longer intervals between the two main meals of the day, there can be a reduction in physical activity and exercise during the day (34). During a summer Ramadan, as in recent years, it is difficult to get around in the heat wave all day except to go to work and shopping in the market. Only the movement to the mosque to fulfill the voluntary evening prayers made in groups during the holy month of Ramadan ≪ *Tarawih* ≫ and/or social gatherings between family and friends allow to maintain a minimum level of daily physical activity throughout the month of Ramadan. Being physically active during Ramadan can help the individual to maintain his/her level of conditioning, as well as better deal with intermittent fast (28). However, excessive physical activity can increase the risk of hypoglycemia and hence should be avoided (10).

## Body Composition

The diet during the holy month of Ramadan should be the same as throughout the year, that is, a healthy and balanced diet from qualitative and quantitative perspectives. It should aim to maintain a constant body mass. In the meta-analysis of Tahapary et al. (35), on the impact of Ramadan fasting on the metabolic profile in the 2TDM patients, a change was found in body weight in 15 studies with 2,511 patients. Overall, there was a slight overall reduction in body weight of 0.71 kg (CI at 95%—.45–0.003 kg). Similarly, six studies involving 2,289 T2DM subjects reported a decrease in waist circumference of 0.62 cm (CI at 95%–1.31–0.08 cm) before and after the fast of Ramadan. As for the effect of Ramadan fasting on the body composition of the diabetic patient, the results were discordant. The researchers Aloulou et al. (36), Khaled and Belbraouet (7), and McEwen et al. (37) reported changes, in other studies some changes were found (16, 38, 39) and for others no change was found (6, 21, 40–43).

## Fasting, Metabolic Changes, Complications, and Clinical Aspects

Fasting induces a secondary adaptive metabolic cascade to hormonal changes. The challenge is to make the best use of stored energy substrates during feeding periods, by keeping blood glucose levels as low as possible in the normal range, while preserving the protein capital (44). Metabolic adaptation to fast is designed to spare fat free mass (mainly muscle mass) or active cell mass. At the initial phase of fasting and in normal conditions, the body mobilizes fat reserves which are oxidized into ketone bodies then used by the brain; at this point, neo-glucogenesis of protein origin is reduced, allowing some saving of the free-fat mass (45). In the case of diabetes, lack of insulin promotes metabolism to ketone body production, which can cause diabetic ketoacidosis. This situation is compounded by difficulties in adjusting therapeutic doses during this month of fasting, which is not without consequence. Indeed, in T1DM there is no endogenous production of insulin by the pancreas. Therefore, insulin intake is entirely dependent on injections. While in the T2DM, endogenous insulin is still secreted, but inadequately or inappropriately, and glucagon is preponderante (46). During Ramadan, fasting-related complications for diabetics can include hyperglycemia, hypoglycemia, acidoketosis, dehydration, or thrombosis (2, 21, 47–51). These consequences are subject in most cases to hospitalization and complete interruption of fasting until the end of the holy month of Ramadan. In the literature, the variation of certain biochemical and clinical parameters, significant or not, contradicted, has been reported in relation to the effect of fasting in diabetics. The study results of Ouhdouch et al. (52) and of Abdessadek et al. (53) reported a significant decrease in HbA1c, while the opposite was noted in the study of Halimi et al. (54) and of Mbainguinam et al. (55) explaining this inequality of HbA1c would be the cause of hypertriglyceridemia. Also, an improvement in the lipid profile (53, 56, 57) was found, a significant decrease in fasting blood glucose during Ramadan compared to outside (53, 57, 58), hyperglycaemic episodes with or without ketoacidosis and severe hypoglycaemia (59–61), the presence of higher hypoglycemia in patients treated with insulin (*p* = 0.002), followed by those treated with oral agents including sulfonylureas as compared to oral agents excluding sulfonylureas (57). Also saved, a non-significant reduction in plasma creatinine, uric acid (53), an improvement in blood pressure figures (57), while in another study no change was found (6, 56).

## COVID-19 Pandemic, Diabetes, and Ramadan Fasting

As for last year, Ramadan fasting took place in a particular context; in the midst of the COVID-19 pandemic. According to international iterim guidelines (62), healthy people should be able to fast during Ramadan, as in previous years, While COVID-19 patients should consider not doing so, following religious exemptions, in consultation with their physician, as with any other illness. According to Khan et al. (63), fasting during the COVID-19 crisis was a challenge for diabetic Muslims during Ramadan. A study of 829 diabetics, 34 fasting patients developed symptoms of COVID-19 before or during Ramadan. Ten patients (four fasters and six non-fasters) were admitted with symptoms related to COVID-19 (nine of whom were confirmed COVID-19 positive), however, none required intensive care. In the study of the DAR 2020 global survey on Ramadan fasting during the COVID-19 pandemic aiming to describe the characteristics and care of participants with T2DM showed that in 5,865 participants (recruited from 20 predominantly Muslim cities) concern over the COVID-19 pandemic affected the decision to fast by 7.6% from ≥65 years old vs. 5.4% from <65 years old, while 94.8% fasted ≥15 days and 12.6% had to break the fast due to diabetes-related illness (64). As reported by Chowdhury et al. (65), if a diabetic develops symptoms of COVID-19 during fasting, he/she must be told to break the fast immediately, to hydrate, to regularly monitor capillary blood glucose, because according to the study of Li et al. (66), there is evidence that ketosis and ketoacidosis were more common in people with diabetes and COVID-19. In their article Chowdhury et al. (65) continued that this applies particularly to patients on sodium-glucose 2 transporter inhibitors (SGLT2i) (67). If a patient is not feeling well with symptoms of COVID-19, it may require hospitalization as it is at high risk of deterioration. There are guidelines suggesting that metformin and SGLT2i should be discontinued in all diabetic and suspected COVID-19 patients requiring hospitalization (65, 68).

## Nutritional Education

Ramadan is a special time in the life of Muslim diabetic patients leading to a major and sudden change in their rhythm of life having an impact on the management of their diabetes (69, 70). In a study highlighting the point of view of health professionals on the difficulties encountered during medical consultations during Ramadan, it was noted: treatment management (69.1%), glycaemic variations (52.1%), venous thrombosis (8.8%), weight gain (2.8%), changes in eating habits (42.9%) and food intake schedules (50.7%). The results of this study also showed that out of 1,975 diabetic patients, 924 of them did not know about the conditions for interruption of fasting whether T1DM or T2DM (53.0 vs. 46.4%; *p* = 0.2984) (13). In parallel, in another study highlighting the role of nutritional education in the management of T2DM during Ramadan, the researchers showed that 96% of the patients who received educational sessions were able to fast more than 21 days with a frequency of hypoglycemia 9 times lower compared to the control group (71). The patient wishing to fast must also be followed nutritionally; pair a balanced and varied diet (coverage of recommended nutritional intakes and respect for food preferences), appropriate treatment (in molecule, dose and frequency of intake), added to glycaemic control, will allow the diabetic patient to better manage his/her disease (13, 69, 70). Pre-Ramadan education is crucial to fast safely during the holy month. Education on fasting and diabetes management is also useful beyond Ramadan (72) having regard to the number of fasters after Ramadan [Example: CREED study (73) between 8 and 46%] and its important place in Muslim society. Admittedly, it is not an obligation, but an accomplishment of a pious act and an inner comfort that can be achieved throughout the year, but also the possibility and not the obligation (especially for the sick patients) to catch up for the unfasted days of the month of Ramadan (13).

## Essentials Points in Term for Recommendations

Several international recommendations and consensus of experts have resulted in proposals to optimize diabetes management during the month of Ramadan:

- The first international recommendations for a good practice of fasting were those of the Hassan II Foundation in Morocco in 1995. This consensus proposed criteria authorizing and prohibiting fasting and reinforced clinical-biological surveillance before, during and after Ramadan, a monitoring of glycemic control, adaptation of treatment, education of diabetics, and their families on the contraindications of fasting, the risk of acute complications and the means of prevention and treatment (74).
- Other recommendations follow those of Morocco, including those of the American Diabetes Association (ADA) published in 2005 (75). In these recommendations, the term “indications” or “counter-indications” of fasting was avoided because the authors considered Ramadan fasting to be a spiritual religious issue for which patients make their own decisions after receiving appropriate advice from religious teachings and their own health professionals. In response to numerous requests and comments on important issues that were not discussed in the previous 2005 document, these recommendations were subsequently revised by regular updates every 5 years and were also the subject of other publications.
- In 2010 (76), research highlighted once again that fast in T1DM patients with poor glycemic control is associated with multiple risks. In the revised document, some issues were discussed, concerning voluntary fasting outside Ramadan, discussion of the effect of prolonged fasting (more than 18 h per day) in remote areas of the equator during Ramadan when it occurs in summer, on the lower risk of hypoglycaemia, such as incretin based therapies and, on the limits of drugs and their use as thiazolidinediones.
- The third update to the recommendations of the ADA Working Group (77) was also published following numerous requests for information on education, dietary habits and new oral and injectable agents that may be useful for the management of diabetic patients during Ramadan.
- In the 2020 update (78), the research group aimed to apply the principles of ADA/European Association for the Study of Diabetes (EASD) type 2 diabetes management guidelines to Ramadan. New sections on the management of T1DM children and gestational diabetes were included. They carefully reviewed the literature and published data on medical nutrition therapy and the use of oral diabetes medication during Ramadan.
- The International Diabetes Federation (IDF) and the Diabetes and Ramadan (DAR) International Alliance met to provide comprehensive guidance on this topic. This guide (79) highlights relevant contextual information and practical recommendations to help diabetic patients to fast during Ramadan while minimizing the risk of complications. The guidelines cover several key topics, including epidemiology, physiology of fasting, risk stratification, nutritional advice, medication adjustment, and implementation of recommendations. One of the recurring themes in the guidelines is the importance of individualization and education as part of a diabetes management plan.
- The update of the IDF-DAR International Alliance Practical Guidelines features new guidance based on a greater and more recent body of evidence (80). This includes an updated set of criteria for risk stratification; information on the impact of fasting on physical and mental wellbeing; specific guidance on the management of T1DM and T2DM in special populations such as pregnant women and the elderly; and information on changes to the risk of comorbidities such as cardiovascular disease, stroke, and renal impairment.
- In addition, the Asian recommendations of the South Asian Consensus Guideline, where researchers (81) indicate that it is possible for people with diabetes to fast safely during Ramadan, but requires careful planning in order to avoid problems that could be serious and have long-term effects. The choice of insulin therapy is decided by the previous therapy that the patient is taking and also the blood glucose profiles. The major objective of insulin therapy during Ramadan is to provide adequate insulin to prevent the post meal (After *Iftar*) hyperglycemia and also prevent hypoglycaemia during the period of fast. With the use of analoges, these objectives may be met more easily.

The importance of the socio-cultural context of the holy month of Ramadan associated with the culturally-religious identity to which the diabetic patient is or wants to adhere are parameters to be included in the decision to fast. The advice of health professionals and members of religion will only reassure the diabetic in the accuracy of their decision. Each patient must be monitored individually, taking into account in particular the evolution of his disease, his therapeutic treatment, his socio-economic level, his level of education, his age, and his family status.

## Conclusion

Fasting is a very sensitive period for the glycemic control of DMF and DMNF patients, requiring multidisciplinary preparation and expert advice. This work gave a brief visualization of the effect of Ramadan fasting on a NCDs which is diabetes. The few studies cited reflecting the various points raised (diet, sleep, physical activity, body composition, metabolic changes, nutrition education) showed heterogeneity of the results found probably due to the number of days of fasting, climatic conditions, cultural variations in eating habits, etc. Some slight advantages were noted. These particularities must be taken into account in the development of any study project on this topic. Effective management of diabetes with regular glycemic control will allow the patient to maintain an appropriate metabolic profile. Education on fasting and diabetes management is useful beyond Ramadan. It is important that recommendations have to be based on the gaps in existing data.

## Author Contributions

MBenc: study design, literature searching, and article draft writing. IS, MBent, and FB: draft revision and literature searching. YB: draft revision. All authors agree to be accountable for the content of the work.

## Conflict of Interest

The authors declare that the research was conducted in the absence of any commercial or financial relationships that could be construed as a potential conflict of interest.

## Publisher's Note

All claims expressed in this article are solely those of the authors and do not necessarily represent those of their affiliated organizations, or those of the publisher, the editors and the reviewers. Any product that may be evaluated in this article, or claim that may be made by its manufacturer, is not guaranteed or endorsed by the publisher.
